# Effects of healthy lifestyles on the association between food security and all-cause mortality risk

**DOI:** 10.1186/s12966-026-01898-w

**Published:** 2026-02-25

**Authors:** Jian Gao, Chuan Li, Huan Chen, Zhi-Hao Li, Dan Liu, Si-Yao Liu, Chen Mao

**Affiliations:** 1 Department of Epidemiology, School of Public Health, Southern Medical University, Guangzhou, 510515 Guangdong China; 2 Department of Laboratory Medicine, Microbiome Medicine Center, Department of Epidemiology, School of Public Health, Zhujiang Hospital, Southern Medical University, Guangzhou, 510515 Guangdong China; 3 National Institute of Health Data Science of China, Southern Medical University, Guangzhou, Guangdong China; 4 St. Jude Children’s Research Hospital, 262 Danny Thomas Place Memphis, Memphis, TN USA; 5 Department of Epidemiology, School of Public Health, National Institute of Health Data Science of China, Southern Medical University, Guangzhou, Guangdong China

**Keywords:** Food security, Lifestyle behaviors, Cohort study, All-cause mortality

## Abstract

**Background:**

Food insecurity is associated with increased mortality risk, but whether this association is modified by lifestyle behaviors in the general adult population remains unclear.

**Methods:**

We analyzed seven NHANES cycles (2005–2018) including 41,161 adults (≥ 18 years). Food security was categorized per USDA guidelines. Four lifestyle factors—smoking status, alcohol consumption, physical activity, and sleep duration (< 6.5, 6.5-<7.5, or ≥ 7.5 h/night)—were assessed via standardized questionnaires, and a healthy lifestyle score (0–4) was constructed by summing these four components. All-cause mortality was determined through linkage to the National Death Index through December 31, 2019. We used Cox proportional hazards models to estimate hazard ratios (HRs) and 95% confidence intervals (CIs) for all-cause mortality across food security categories and by healthy lifestyle score, adjusting for demographic and socioeconomic characteristics, health status indicators (BMI, history of diabetes, hypertension, hypercholesterolemia, and health insurance coverage), and dietary factors (total daily energy intake and overall diet quality). We conducted prespecified subgroup analyses by age (< 65 vs. ≥ 65 years), sex, and health insurance status, and performed sensitivity analyses.

**Results:**

During 204,944 person-years of follow-up, 2835 total deaths occurred. Food insecurity was associated with higher all-cause mortality (HR 1.85 [95% CI, 1.60–2.14]; *P* < 0.001). In joint analyses restricted to food-insecure adults, compared with those with the most unfavorable behaviors, participants who never smoked had a 51% lower hazard of mortality (HR, 0.49 [95% CI, 0.39–0.61]), those who never drank alcohol had a 27% lower hazard (HR, 0.73 [95% CI, 0.56–0.97]), those with moderate/regular physical activity had a 32% lower hazard (HR, 0.68 [95% CI, 0.49–0.96]), and those reporting sleep duration of 6.5-<7.5 h/night had a 26% lower hazard (HR, 0.74 [95% CI, 0.55–0.98]). The healthy lifestyle score showed a graded inverse association, among food-insecure adults, having 3–4 versus 0 healthy behaviors was associated with a 61% lower hazard of death (HR, 0.39 [95% CI, 0.23–0.66]). Associations were stronger among participants aged < 65 years and were robust in sensitivity analyses.

**Conclusion:**

Food insecurity is significantly associated with higher all-cause mortality risk, while healthy lifestyle behaviors can effectively buffer this risk, especially in food-insecure adults.

**Supplementary Information:**

The online version contains supplementary material available at 10.1186/s12966-026-01898-w.

## Introduction

Household food security is an essential determinant of health and influences nutritional status and long-term health outcomes. Food insecurity is defined as limited or uncertain access to adequate food and has been associated with an increased risk of chronic disease and premature death [1, 2]. Even marginal food insecurity could lead to a 50% higher risk of early death and a 2.6-year reduction in life expectancy, underscoring its critical role as a socioeconomic health determinant [3, 4].

While the association between food insecurity and mortality is well-established, the mechanisms remain unclear. Behavioral and physiological factors, such as sleep duration, smoking, alcohol use, and physical activity, may mediate this relationship by influencing metabolic regulation, immune function, and stress responses. These behaviors are modifiable determinants of cardiometabolic health and longevity and may plausibly modify the food insecurity-mortality association through pathways involving metabolic regulation, inflammation/immune function, and stress responses [5–8]. Prior epidemiologic studies have consistently reported higher mortality risk among food-insecure adults, but lifestyle behaviors were typically treated as confounders rather than being evaluated as effect modifiers or in joint exposure models, leaving the extent to which healthier behaviors attenuate food insecurity-related mortality insufficiently characterized [9, 10]. Addressing food insecurity directly is challenging due to structural and economic barriers, but promoting healthier behaviors offers a practical approach to mitigating its impacts. However, whether modifying these behaviors reduces food insecurity-related mortality remains uncertain.

This cohort study used nationally representative data from the National Health and Nutrition Examination Survey (NHANES) linked to mortality records to evaluate whether sleep duration, smoking, alcohol consumption, and physical activity modified the association between food insecurity and all-cause mortality. The findings aim to identify actionable behavioral strategies to reduce mortality risk in food-insecure populations and inform targeted interventions addressing health disparities.

## Methods

### Study design and participants

This cohort study included data on adults aged 18 years or older who completed the US Department of Agriculture (USDA) Adult Food Security Survey Module during the 7 cycles of the NHANES from 2005 to 2006 to 2017–2018. The NHANES study design has been described in detail previously [11]. After excluding participants with missing food security data (*n* = 885) or incomplete mortality records (*n* = 121), the final analytic sample comprised 41,161 individuals (eFigure 1). The National Center for Health Statistics Institutional Review Board approved all study protocols, with written informed consent obtained from participants prior to data collection.

### Assessments of exposure

Food insecurity was assessed using the 10-item USDA Adult Food Security Survey Module [12] (eTable 1), Household food security was categorized into four levels: full (0 affirmative responses), marginal (1–2), low (3–5), and very low security (6–10). We additionally used a binary classification for readability, defining participants with full security as food secure and those with marginal/low/very low security as food insecure. The validity of this module has been confirmed in diverse racial and ethnic populations [13].

Four key lifestyle factors were assessed: smoking status (never/former/current smoker), alcohol consumption (never/former/current drinker), physical activity level (vigorous/moderate/sedentary), and self-reported sleep duration. Sleep duration was categorized as < 6.5 h, 6.5-<7.5 h, and ≥ 7.5 h/night. We used ~ 7 h/night as the anchor, as it is associated with the lowest all-cause mortality risk group [14–18]. Accordingly, 6.5-<7.5 h/night (closest to 7 h) was defined as the optimal sleep group. Sleep disturbance was recorded dichotomously (yes/no).

Comprehensive covariates included demographic characteristics (age, gender, race/ethnicity, education, income, household size), body mass index (BMI), chronic conditions (self-reported history of diabetes, hypertension, and hypercholesterolemia), and health insurance coverage, collected via standardized NHANES interviews, and total daily energy intake. Overall diet quality was assessed using the Healthy Eating Index-2015 (HEI-2015) derived from NHANES dietary recall data and was included in sensitivity analyses.

### Assessments of outcome

The corresponding mortality information for each participant was identified through linkage to the National Death Index up to 31 December 2019. For this study, the primary outcome was all-cause mortality. The International Classification of Diseases (ICD)-10 was used to determine disease-specific deaths.

### Statistical analysis

All analyses incorporated NHANES sampling weights, strata, and primary sampling units to account for the complex survey design [19]. Baseline characteristics were summarized in the overall population and across categories of food security status. Continuous variables were presented as weighted means with standard errors (SEs), and categorical variables as unweighted counts with weighted percentages. Primary results are based on a single prespecified fully adjusted model, in which covariates were entered simultaneously. The fully adjusted cox regression model included sociodemographic factors (age, sex, and race/ethnicity), socioeconomic indicators (education, family income, and household size), and health- and lifestyle-related factors (BMI, health insurance coverage, self-reported physician-diagnosed diabetes, hypertension, and hypercholesterolemia/dyslipidemia, and total daily energy intake). When individual lifestyle factors were examined as the exposure, the model additionally included food security and the remaining lifestyle factors to achieve mutual adjustment. The proportional hazards assumption was evaluated using Schoenfeld residuals.

To evaluate whether combined adherence to healthy behaviors modified the association between food security and mortality, we constructed a healthy lifestyle score based on four behaviors: non-smoking, non-drinking, regular physical activity, and sleep duration in the reference range (6.5-<7.5 h). One point was assigned for each healthy behavior (range 0–4), and participants were categorized as 0, 1, 2, or 3–4 healthy behaviors.

We formally tested multiplicative interaction by including cross-product terms between food insecurity and each lifestyle factor (and the lifestyle score) in the fully adjusted models; P-interaction values were obtained from Wald tests. Stratified analyses examined effect modification by gender, age (< 65 vs. ≥65 years), and insurance status. Sensitivity analyses addressed potential reverse causation (excluding participants with < 2 years follow-up, *n* = 35062), confounding by chronic conditions(*n* = 15017), sleep disturbances(*n* = 41161), extreme caloric intake (< 500/>5000 kcal/day. *n* = 40727), and overall diet quality using HEI-2015 (*n* = 41161).

All analyses were conducted using R (version 4.0.5), with statistical significance set at two-tailed *p* < 0.05.

### Patient and public involvement statement

NHANES participants provided informed consent but were not involved in study design or implementation. Results will be disseminated through cohort websites and public forums. Study funders had no role in the research process.

## Results

### Participant characteristics

A total of 41 161 adults were included in the study (weighted mean [SE] age, 46.45 [0.23] years, 51.8% female, 9060 Black individuals [11.5%], 6601 Mexican individuals [8.6%], 3900 Other Hispanic individuals [5.5%], 16 952 White individuals [66.6%], and 4648 individuals of other races, including multiracial [7.7%]). Characteristics of the sample with weighted population numbers are presented in Table 1. Of the participants, 76.4% had full food security; 9.4% of participants had marginal food security; 8.1% of participants had low food security; and 6.1% of participants had very low food security.

**Table 1:** Baseline characteristics of participants according to food security status

Characteristic	Total participants	Food security	Food insecurity
Food Security Category, *n* (%)			
Full security	28,303 (76.4)	28,303(100)	-
Marginal security	5014 (9.42)	-	5014(39.94)
Low security	4604 (8.05)	-	4604(34.14)
Very low security	3240 (6.12)	-	3240 (25.92)
Mean age, *y(SE)*	46.45 (0.23)	47.99 (0.25)	41.48 (0.27)
Body Mass Index,*kg/m2 (SE)*	28.92 (0.08)	28.62 (0.09)	29.86 (0.12)
Gender, *n (%)*			
Male	19,983 (48.21)	13,982 (48.81)	6001 (46.26)
Female	21,178 (51.79)	14,321 (51.19)	6857 (53.74)
Race/Ethnics, *n (%)*			
Mexican American	6601 (8.61)	3565 (6.03)	3036 (16.97)
Other Hispanic	3900 (5.53)	2235 (4.11)	1665 (10.09)
Non-Hispanic White	16,952 (66.62)	13,271 (72.78)	3681 (46.7)
Non-Hispanic Black	9060 (11.51)	5717 (9.35)	3343 (18.49)
Other Race	4648 (7.73)	3515 (7.72)	1133 (7.75)
Education level, *n (%)*			
Less than 9th grade	4263 (5.44)	2149 (3.65)	2114 (11.23)
9−11th grade	5673 (10.44)	3241 (8.33)	2432 (17.29)
High school	9081 (22.79)	5958 (21.28)	3123 (27.67)
College	11,394 (30.32)	7996 (30.19)	3398 (30.73)
College graduate or above	8716 (28.17)	7731 (34.00)	985 (9.28)
Annual household income, *n (%)*			
Under $20,000	15,179 (30.99)	8141 (24.12)	7038 (53.21)
$20,000 to $45,000	10,655 (25.63)	7262 (25.53)	3393 (25.98)
$45,000 to $75,000	5425 (14.79)	4365 (16.33)	1060 (9.80)
$75,000 and Over	9831 (28.49)	8492 (33.95)	1339 (10.83)
Household Size, *n (%)*			
1	5711 (13.14)	4157 (13.49)	4157 (12.01)
2	12,054 (33.01)	9346 (36.29)	9346 (22.40)
3	7447 (18.67)	5160 (18.49)	5160 (19.26)
4	6724 (17.26)	4561 (16.95)	4561 (18.27)
5	4490 (9.85)	2708 (8.84)	2708 (13.10)
6	2324 (4.32)	1254 (3.39)	1254 (7.33)
7 or more	2411 (3.75)	1117 (2.55)	1117 (7.63)
Diabetes, *n (%)*			
Yes	5025 (9.20)	3272 (8.67)	1753 (10.93)
No	36,110 (90.75)	25,018 (91.28)	11,092 (89.01)
Hypertension, *n (%)*			
Yes	14,053 (30.80)	9788 (31.00)	4265 (30.17)
No	27,045 (69.08)	18,482 (68.92)	8563 (69.61)
Cholesterol, *n (%)*			
Yes	12,660 (30.87)	9183 (32.46)	3477 (25.75)
No	21,690 (55.46)	14,962 (55.05)	6728 (56.77)
Health Insurance Covered, *n (%)*			
Yes	32,300 (82.28)	23,868 (87.19)	8432 (66.35)
No	8787 (17.60)	4398 (12.70)	4389 (33.44)
Smoking Status, *n (%)*			
Current	8039 (19.82)	4422 (16.07)	3617 (31.96)
Quitting	9350 (23.69)	7007 (25.38)	2343 (18.22)
Never	22,276 (54.54)	15,955 (56.77)	6321 (47.31)
Alcohol consumption, *n (%)*			
Current	25,751 (69.46)	17,812 (69.97)	7939 (67.82)
Quitting	5268 (9.99)	3446 (9.43)	1822 (11.79)
Never	4002 (8.34)	2850 (8.52)	1152 (7.78)
Physical Activity Status, *n (%)*			
Sedentary activity	17,361 (38.42)	11,632 (37.05)	5729 (42.85)
Moderate Activity	17,691 (47.78)	13,170 (51.24)	4521 (36.58)
Vigorous Activity	6107 (13.80)	3501 (11.71)	2606 (20.57)
Sleep Duration Hours,*h (SE)*	7.13 (0.01)	7.15 (0.01)	7.08 (0.02)
Sleep Duration Category, *n (%)*			
Less than 6.5	13,412 (29.95)	8837 (28.45)	4575 (34.82)
6.5 and over, and less than 7.5	11,408 (31.07)	8290 (32.62)	3118 (26.02)
7.5 and over	16,214 (38.98)	11,105 (38.93)	5109 (39.16)

^a^Continuous variables were presented as weighted mean (95% CI), and categorical variables were presented as unweighted number of participants (weighted percentage)

Compared with food-secure participants, food-insecure participants were younger (mean age 41.48 vs. 47.99 years) and had a higher mean BMI (29.86 vs. 28.62 kg/m²). They were more frequently non-Hispanic Black (18.49% vs. 9.35%), Mexican American (16.97% vs. 6.03%), or other Hispanic (10.09% vs. 4.11%), and were more likely to have lower socioeconomic status (annual household income <$20,000: 53.21% vs. 24.12%; college graduate or above: 9.28% vs. 34.00%). Food-insecure participants were less likely to have health insurance coverage (66.35% vs. 87.19%) and more likely to be current smokers (31.96% vs. 16.07%) or former drinkers (11.79% vs. 9.43%). Regarding sleep, short sleep duration (< 6.5 h) was more common in the food-insecure group (34.82% vs. 28.45%), whereas 6.5-<7.5 h was less common (26.02% vs. 32.62%). Self-reported diabetes was slightly more prevalent among food-insecure participants (10.93% vs. 8.67%).

## Association between food security and all-cause mortality

**Fig. 1:**
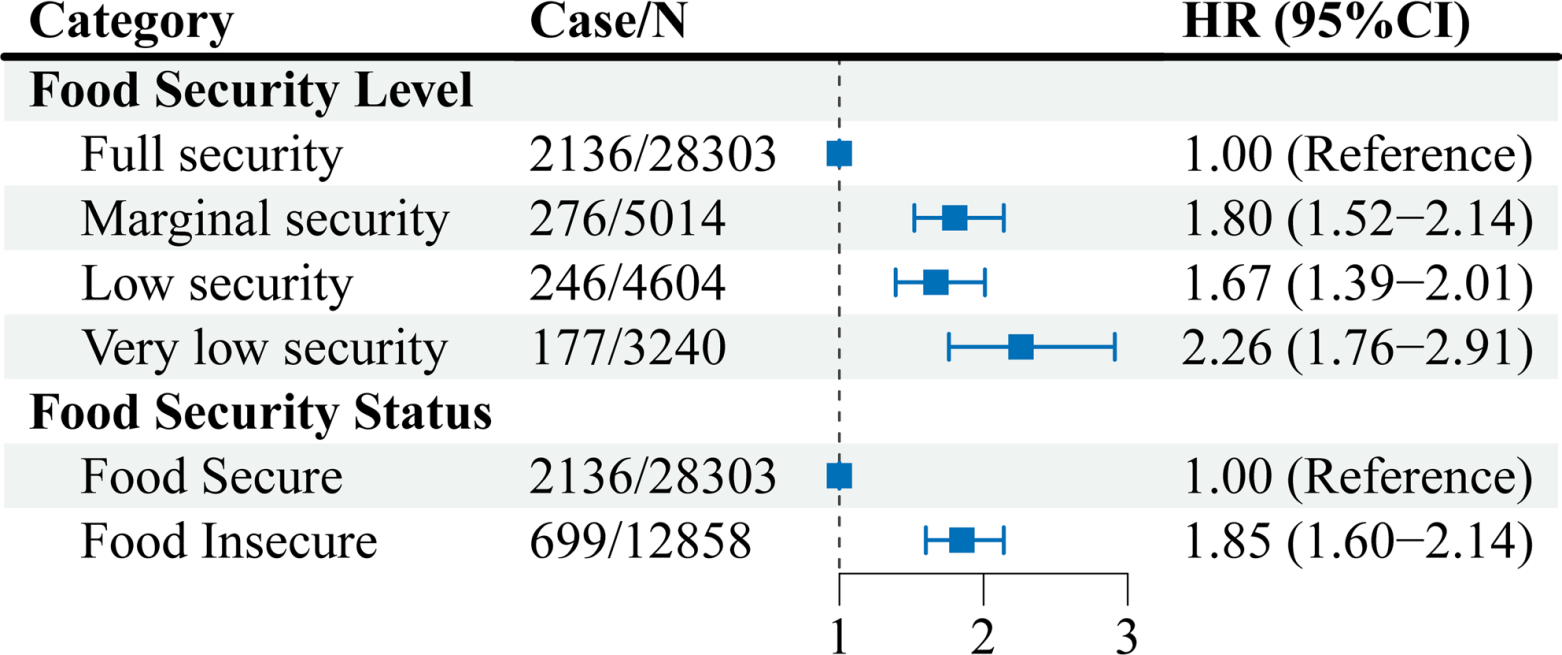
Associations of food safety categories and all-cause mortality risk. Multivariate Cox regression models included US population and study design weights to account for the complex survey design. Hazard ratios were adjusted for age, gender, race/ethnicity, education level, annual household income, household size, BMI, disease history of diabetes, hypertension, and hypercholesterolemia, daily energy intake, health insurance coverage, and lifestyles factors, including regular physical activity, current smoking status, current drinking status, and sleep duration. The squares represent hazard ratios. The solid lines represent 95% confidence intervals

We found participants with food insecurity compared with those with food security had a 85% higher risk of all-cause mortality (HR, 1.85 [95% CI, 1.60–2.14]). When food security was further classified into four levels, compared with participants with full food security, participants with marginal food security, low food security, and very low food security had 80%, 67%, and 126% higher hazards of all-cause mortality, respectively (HR, 1.80 [95% CI, 1.52–2.14], HR, 1.67 [95% CI, 1.39–2.01] and HR, 2.26 [95% CI, 1.76–2.91]) (Fig. 1; eTable 2).

### The association between food security and all-cause mortality risk across different healthy lifestyle scores

**Fig. 2:**
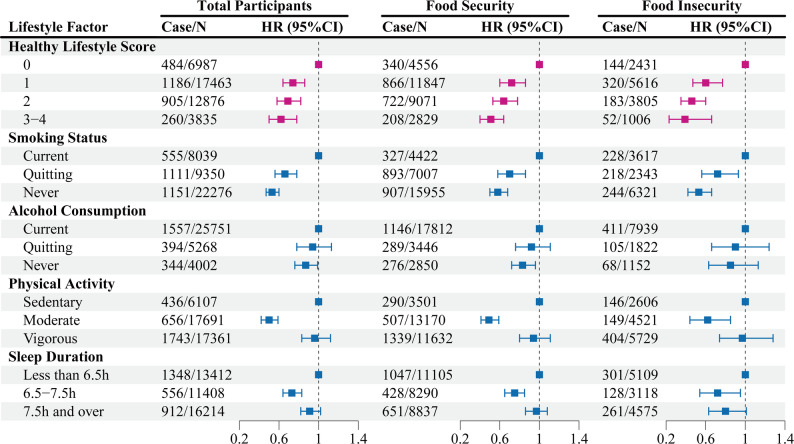
Association of lifestyle factors and healthy lifestyle scores with risk of all-cause mortality according to food security status. Multivariate Cox regression models included US population and study design weights to account for the complex survey design. Hazard ratios were adjusted for age, gender, race/ethnicity, education level, annual household income, household size, BMI, disease history of diabetes, hypertension, and hypercholesterolemia, daily energy intake, health insurance coverage, and lifestyles factors, including regular physical activity, current smoking status, current drinking status, and sleep duration. The squares represent hazard ratios. The solid lines represent 95% confidence intervals

We examined the association across categories of the healthy lifestyle score (0, 1, 2, and 3–4). Among individuals with food insecurity, having 1, 2, and 3 to 4 healthy lifestyle factors was associated with a 40% (HR, 0.60 [95% CI, 0.47–0.77]), 54% (HR, 0.46 [95% CI, 0.35–0.60]), and 61% (HR, 0.39 [95% CI, 0.23–0.66]) lower risk of all-cause mortality, respectively, compared with those with no healthy lifestyle factors. Notably, among individuals with food security, the corresponding reductions in all-cause mortality risk were 28% (HR, 0.72 [95% CI, 0.60–0.86]), 36% (HR, 0.64 [95% CI, 0.53–0.78]), and 49% (HR, 0.51 [95% CI, 0.40–0.64]), respectively (Fig. 2, eTable 3).

### Association of lifestyle factors and risk of all-cause mortality among different food security participant

Among participants with food security, four individual lifestyle factors were significantly associated with all-cause mortality. Using current smoking, current drinking, vigorous physical activity, and a sleep duration longer than 7.5 h as reference categories, quitting smoking (HR, 0.70 [95% CI, 0.58–0.86]) and never smoking (HR, 0.58 [95% CI, 0.50–0.68]) were associated with a lower risk of all-cause mortality. Similarly, never drinking (HR, 0.83 [95% CI, 0.72–0.96]), engaging in moderate physical activity (HR, 0.49 [95% CI, 0.41–0.59]), and reporting a sleep duration between 6.5 and 7.5 h (HR, 0.75 [95% CI, 0.65–0.85]) were also associated with reduced mortality risk (Fig. 2, eTable 4).

In addition, among the food insecurity group, all-cause mortality risk was also influenced by the four lifestyle factors. Compared with current-smoking, quitting-smoking (HR, 0.72 [95% CI, 0.56–0.93]) and never-smoking (HR, 0.53 [95% CI, 0.42–0.66]) were associated with a 28% and 47% lower risk of all-cause mortality, respectively. Compared with current-drinking, both quitting-drinking and never-drinking showed a trend toward lower all-cause mortality risk, though not statistically significant. Compared with vigorous activity, moderate activity (HR, 0.62 [95% CI, 0.44–0.85]) was associated with a 38% lower risk. Compared with a sleep duration longer than 7.5 h, a sleep duration between 6.5 and 7.5 h (HR, 0.72 [95% CI, 0.54–0.95]) was associated with a 28% lower risk of all-cause mortality (Fig. 2, eTable 4).

### Joint analysis of lifestyle and food security with all-cause mortality

We further assessed the joint associations of food security status and lifestyle behaviors with all-cause mortality risk. Compared with individuals experiencing food insecurity combined with the most unfavorable lifestyle factors—including current smoking, current drinking, vigorous physical activity, and long sleep duration—those with food security consistently exhibited significantly lower all-cause mortality risk across all lifestyle categories.

**Fig. 3:**
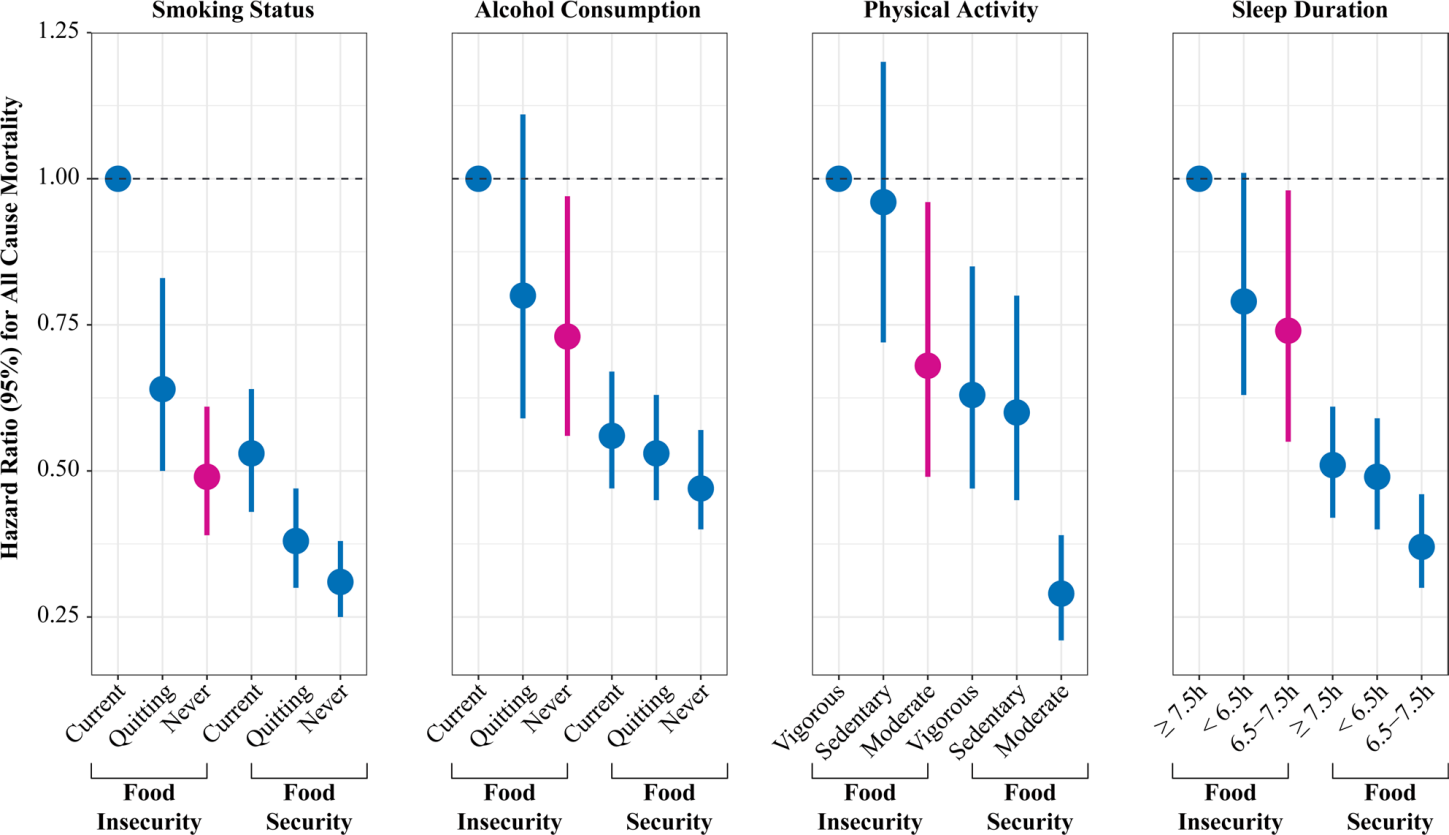
Associations of lifestyle factors with mortality by food security status. Multivariate Cox regression models included US population and study design weights to account for the complex survey design. Hazard ratios were adjusted for age, gender, race/ethnicity, education level, annual household income, household size, BMI, disease history of diabetes, hypertension, and hypercholesterolemia, daily energy intake, health insurance coverage, and lifestyles factors, including regular physical activity, current smoking status, current drinking status, and sleep duration. Blue dots and lines represented the HR (95% CI) of smoking status. Yellow dots and lines represent the HR (95% CI) of alcohol consumption. Green dots and lines represent the HR (95% CI) of physical activity. Pink dots and lines represent the HR (95% CI) of sleep duration

Among food-insecure individuals, only those adopting healthier lifestyle behaviors demonstrated significant reductions in mortality risk. Specifically, within the food-insecure group, individuals who had quit smoking (HR, 0.64 [95% CI, 0.50–0.83]) or had never smoked (HR, 0.49 [95% CI, 0.39–0.61]) showed 36% and 51% reductions in mortality risk, respectively, compared with current smokers. Regarding alcohol consumption, never-drinkers demonstrated a 27% lower mortality risk (HR, 0.73 [95% CI, 0.56–0.97]) compared with current drinkers. For physical activity, individuals engaging in moderate activity had a 32% reduction in mortality risk (HR, 0.68 [95% CI, 0.49–0.96]) compared with those with vigorous activity levels(Fig. 3, eTable5). In terms of sleep duration, individuals with a sleep duration between 6.5 and 7.5 h had a 26% lower risk of all-cause mortality (HR, 0.74 [95% CI, 0.55–0.98]) compared with those with longer sleep duration.

### Subgroup and sensitivity analyses

We further conducted stratified analyses by gender, age, and health insurance status. No significant interactions were observed for gender (*P* = 0.050) or health insurance (*P* = 0.793), whereas a significant interaction was identified for age (*P* = 0.013). The inverse association between healthy lifestyle and all-cause mortality was more pronounced among participants younger than 65 years old. Nonetheless, across all strata, compared with individuals with food insecurity and unhealthy lifestyles, both food-secure individuals and those with food insecurity but healthy lifestyles consistently showed a reduced risk of all-cause mortality(eTable6-8). In the fully adjusted models, we also observed statistically significant multiplicative interactions between food insecurity and sleep duration (*P*-interaction < 0.001), smoking status (*P*-interaction = 0.041), alcohol consumption (*P*-interaction = 0.032), physical activity (*P*-interaction = 0.001), and the lifestyle score (*P*-interaction = 0.003) (eTable 9).

In sensitivity analyses, excluding participants with less than two years of follow-up, those with chronic diseases (including hypertension, diabetes, and hyperlipidemia), those who had abnormal caloric intake, and additionally adjusting for sleep-related factors and HEI-2015, the results remained robust. The protective association between food security, healthy lifestyle factors, and reduced mortality risk persisted across all models(eTable10-14).

## Discussion

This study, based on nationally representative prospective cohort data, systematically evaluated the association between household food security status and all-cause mortality. To our knowledge, this study is the first to find that adherence to a healthy lifestyle, especially never smoking and drinking, moderate physical activity and optimal sleep time, significantly reduces the risk of death even among people with food insecurity. Importantly, we observed a clear dose-response relationship between the number of healthy lifestyle factors and mortality risk. Among individuals with food insecurity, those adopting 3–4 healthy behaviors experienced a 61% reduction in mortality risk (HR = 0.39), which was even greater than the 49% reduction (HR = 0.51) observed among food-secure individuals. This suggests that healthy lifestyle adherence may partially offset the elevated mortality risk associated with food insecurity. These findings underscore the dual necessity of addressing structural drivers of food insecurity while promoting modifiable behaviors to mitigate health disparities.

Food insecurity remains a major public health challenge with wide-ranging implications for individuals, families, and society. Addressing it requires sustained and coordinated efforts at the policy level, including long-term investments in food systems, social protection, and structural support. At the same time, encouraging individuals to reduce health risks through modifiable factors offers a practical and complementary approach, particularly in the face of persistent resource constraints. To support such efforts, the findings of this study provide timely evidence to inform targeted and scalable prevention strategies.

While previous research has consistently linked food insecurity to elevated mortality risk, our study contributes new insights in three critical ways [10]. First, we identified a graded relationship between food security status and all-cause mortality, which remained robust after adjusting for a wide array of sociodemographic, clinical, and behavioral confounders. Second, although earlier studies have often treated lifestyle factors as potential confounders [9], we demonstrated their active moderating role—showing that even partial adherence to healthy behaviors (1–2 practices) reduced mortality risk by 40%-54% in food-insecure populations. Third, our findings are consistent with potential biological mechanisms reported in prior literature through which healthy behaviors may mitigate the adverse health consequences of food insecurity, for example, improved metabolic regulation associated with physical activity [20–22] and reduced exposure to oxidative stressors associated with smoking avoidance/cessation [23–25]. However, as intermediate biomarker data (e.g., oxidative stress markers) were not available and lifestyle indicators served as pragmatic, non-invasive proxies, the mechanistic implications of our findings should be interpreted with caution and considered hypothesis-generating; importantly, such lifestyle-based proxies are readily obtainable and scalable in large populations, offering practical public health utility when direct biomarker assessment is not feasible. These insights challenge the notion that socioeconomic determinants wholly overshadow behavioral interventions, instead advocating for integrated approaches.

The mechanisms underlying the mortality burden of food insecurity are multifactorial. Chronic stress, suboptimal nutrition, and limited health care access may synergistically exacerbate cardiometabolic dysfunction, systemic inflammation, and mental health disorders [2, 26–33]. For instance, food-insecure individuals experience cortisol dysregulation amplified by financial strain, which impairs glycemic control and immune responses [1, 34, 35]. Concurrently, poor dietary quality—characterized by energy-dense, nutrient-poor food choices—accelerates the development of obesity and hypertension [36–39]. Furthermore, the sustained psychological and physiological stress imposed by food insecurity may promote metabolic dysregulation and disordered eating behaviors, thereby amplifying the burden of chronic diseases and mortality risk [40–43].

The mechanisms by which lifestyle behaviors mitigate the adverse effects of food insecurity on health are multifaceted. Regular moderate-intensity physical activity significantly improves metabolic abnormalities commonly seen in food-insecure individuals, such as insulin resistance, lipid metabolism disorders, and chronic inflammation [26, 27, 44–46]. Exercise enhances insulin sensitivity, regulates inflammation, and improves energy utilization, reducing the risk of cardiovascular disease, type 2 diabetes, and obesity-related mortality [7, 47–49]. Our study found that even in food-insecure populations, those who maintained regular physical activity had a significantly lower risk of all-cause mortality. Additionally, restricting tobacco intake has been shown to reduce inflammation and immune dysregulation, which are particularly pronounced in the context of food insecurity and chronic stress. Smoking exacerbates oxidative stress and impairs immune function [5, 50], effects that are amplified in food-insecure individuals who are often nutrient-deficient. By avoiding smoking, individuals reduce oxidative burden and inflammatory responses, which may preserve immune function and decrease the risk of cardiovascular disease and cancer. Furthermore, limiting alcohol consumption is critical for food-insecure populations, as excessive drinking exacerbates malnutrition, depletes essential nutrients, and increases the risk of anxiety and depression [6, 51, 52]. Our findings indicate that individuals who limited alcohol intake had a significantly lower mortality risk across all levels of food security. Adequate sleep is crucial for regulating stress and maintaining metabolic health. Food insecurity increases psychological stress, which adversely affects sleep quality and structure, further exacerbating cardiovascular and metabolic risks [53–55]. Both insufficient sleep and excessive sleep can have harmful effects: insufficient sleep raises cortisol levels, decreases insulin sensitivity [8, 56], and activates the sympathetic nervous system, while excessive sleep may signal underlying health issues such as malnutrition or depression [57, 58]. Our study found that moderate sleep duration, defined as 6.5–7.5 h per night, helps buffer the adverse effects of food insecurity by mitigating stress-induced metabolic disturbances and lowering mortality risk. These findings emphasize the importance of maintaining an optimal sleep duration to protect against the health consequences of food insecurity. Moreover, by constructing a healthy lifestyle score, we observed that adherence to more healthy lifestyle behaviors, even among food-insecure individuals, was associated with significantly lower mortality risk, emphasizing the importance of lifestyle interventions in these vulnerable populations.

These findings carry urgent policy implications. While systemic reforms—such as expanding nutrition assistance programs and living wage policies—remain imperative, healthcare systems must concurrently prioritize lifestyle interventions tailored to food-insecure populations. Clinicians should screen for food insecurity during routine visits and link patients to behavioral support (e.g., smoking cessation programs, sleep hygiene resources). Community-level partnerships, such as subsidized fitness initiatives and mobile health coaching, could enhance accessibility. Public health campaigns emphasizing incremental behavior changes (e.g., “even small steps matter”) may empower high-risk groups to adopt sustainable practices.

Stratified analyses by gender, age, and health insurance status showed that the inverse association between multiple healthy lifestyle behaviors and all-cause mortality among food-insecure individuals persisted across all subgroups. Although some interaction terms reached statistical significance, the mortality risk was consistently lower among individuals engaging in more healthy behaviors, regardless of gender, age group, or insurance coverage, suggesting the widespread benefits of lifestyle modification.

Moreover, sensitivity analyses further strengthened the inference of causality. After excluding participants with less than two years of follow-up, the associations remained robust, mitigating concerns of reverse causation. Importantly, when individuals with preexisting chronic conditions—such as hypertension, diabetes, and hyperlipidemia—were excluded, the association between multiple healthy lifestyles and reduced mortality still held, suggesting that the observed benefits are not entirely mediated by baseline disease status. Additionally, after adjusting for sleep-related disorders, the protective effects of healthy lifestyle behaviors remained significant, underscoring their independent contribution beyond sleep health. Finally, after excluding participants who failed to respond appropriately to food and nutrition-related questionnaires, we still observed the effect of healthy lifestyle on the relationship between food security and mortality. Together, these findings reinforce the potential of lifestyle-based strategies as effective interventions for reducing mortality risk, even in high-risk populations such as those experiencing food insecurity. These results highlight the need for integrated public health approaches that promote healthy behaviors while addressing structural determinants like food access and healthcare equity.

This study has several strengths, including the use of a nationally representative large sample, a long time span, detailed lifestyle measurements, systematic stratification and interaction analyses, and rigorous sensitivity testing. However, it also has limitations. First, food security and lifestyle behaviors were self-reported, which may introduce measurement error. Second, exposures were assessed at baseline only; food security status (reflecting the prior 12 months) may change over time due to employment, health, or household circumstances, and such non-differential misclassification would likely attenuate associations toward the null, potentially underestimating the true effect. Third, although we adjusted for a wide range of confounders, residual confounding cannot be completely excluded.

## Conclusion

Food insecurity is strongly associated with increased all-cause mortality, yet this risk can be substantially attenuated through adherence to healthy lifestyle behaviors. Notably, even among food-insecure individuals, those engaging in multiple healthy practices experienced mortality reductions surpassing those observed in food-secure counterparts. These findings reveal the powerful compensatory role of lifestyle in offsetting socioeconomic disadvantage and underscore a critical opportunity: future public health strategies must go beyond addressing food access alone and systematically embed behavior-based interventions to enhance resilience and reduce health disparities among vulnerable populations.

## Supplementary Information

Supplementary Material 1.

## Data Availability

The raw data supporting the conclusions of this article will be made available by the authors, without undue reservation. Study protocol and data set: Available at wwwn.cdc.gov/nchs/nhanes. Statistical code: Available from Dr. Gao (e-mail, gaojian_2022@smu.edu.cn).

## Abbreviations

NHANES: National Health and Nutrition Examination Survey
USDA: US Department of Agriculture
HEI-2015: Healthy Eating Index-2015
ICD: International Classification of Diseases
SEs: Standard errors
HRs: Hazard ratios
CIs: Confidence intervals
