# Supplementary material for: Effects of healthy lifestyles on the association between food security and all-cause mortality risk
Source: Int J Behav Nutr Phys Act. 2026 Feb 25;23:32. doi: 10.1186/s12966-026-01898-w (PMC13040838; doi:10.1186/s12966-026-01898-w)
Supplement: Supplementary file 1 — Supplementary Material 1. [file 12966_2026_1898_MOESM1_ESM.docx]

**Effects of Healthy Lifestyles on the Association Between Food Security and All-Cause Mortality Risk**

[eTable 1. US Department of Agriculture Adult Food Security Survey Module Questions 2](#_Toc5967)

[eTable 2. Adjusted Hazard Ratios for All-Cause Mortality by Food Security Status 4](#_Toc26723)

[eTable 3. Adjusted Hazard Ratios of All-cause Mortality Associated with Different Lifestyles Scores and Food Security Status 5](#_Toc22860)

[eTable 4. Stratified Hazard Ratios for All-Cause Mortality Associated with Individual Lifestyle Factors 6](#_Toc19157)

[eTable 5. All-Cause Mortality Hazard Ratios from Joint Effects of Lifestyle Factors and Food Security Status 8](#_Toc5984)

[eTable 6. All-cause Mortality Hazard Ratios Stratified by Gender 11](#_Toc17528)

[eTable 7. All-cause Mortality Hazard Ratios Stratified by Age 13](#_Toc25205)

[eTable 8. All-cause Mortality Hazard Ratios Stratified by Health Insurance Status 15](#_Toc16476)

[eTable 10. Hazard Ratios for All-Cause Mortality by Food Security Status and Lifestyle After Excluding Participants with Follow-Up Less Than 2 Years 18](#_Toc18432)

[eTable 11. Hazard ratios for All-Cause Mortality by Food Security Status and Lifestyle After Excluding Chronic Diseases 20](#_Toc12861)

[eTable 12. Hazard ratios for All-Cause Mortality by Food Security Status and Lifestyle After Additional Adjustment for Sleep-related Factors 22](#_Toc122)

[eTable 13. Hazard ratios for All-Cause Mortality by Food Security Status and Lifestyle After Additional Adjustment for Energy-related Factors 24](#_Toc32151)

[eTable 14. Hazard ratios for All-Cause Mortality by Food Security Status and Lifestyle After Additional Adjustment for diet quality using the Healthy Eating Index-2015 26](#_Toc25267)

[eFigure 1. Flow Chart of Participants Enrolment 28](#_Toc8757)

# **eTable 1. US Department of Agriculture Adult Food Security Survey Module Questions**

|  | **Now I'm going to read you several statements that people have made about their food situation. Please tell me whether the statement was often, sometimes, or never true in the last 12 months.** | **Responses** |
| --- | --- | --- |
| **Q1** | “I worried whether our food would run out before we got money to buy more.” | Often True  Sometimes True  Never True |
| **Q2** | “The food that we bought just didn't last, and we didn't have money to get more.” | Often True  Sometimes True  Never True |
| **Q3** | “We couldn't afford to eat balanced meals.” | Often True  Sometimes True  Never True |
| **Q4** | In the last 12 months, did you or other adults in the household ever cut the size of your meals or skip meals because there wasn't enough money for food? | Yes or no |
| **Q5** | (If yes to question 4) How often did this happen-almost every month, some months but not every month, or in only 1 or 2 months? | Almost every month  Some months but not every month  Only 1 or 2 months |
| **Q6** | In the last 12 months, did you ever eat less than you felt you should because there wasn't enough money for food? | Yes or no |
| **Q7** | In the last 12 months, were you ever hungry, but didn't eat, because there wasn't enough money for food? | Yes or no |
| **Q8** | In the last 12 months, did you lose weight because there wasn't enough money for food? | Yes or no |
| **Q9** | In the last 12 months did you or other adults in your household ever not eat for a whole day because there wasn't enough money for food? | Yes or no |
| **Q10** | (If yes to question 9) How often did this happen? | Almost every month  Some months but not every month  Only 1 or 2 months |

# **eTable 2. Adjusted Hazard Ratios for All-Cause Mortality by Food Security Status**

| **Category** | **N** | **All-cause Mortality** | | |
| --- | --- | --- | --- | --- |
|  |  | **Case** | **HR (95% CI)** | **P value** |
| **Food Security Level** | | | | |
| Full security | 28303 | 2136 | 1 (Reference) |  |
| Marginal security | 5014 | 276 | 1.80 (1.52-2.14) | <0.001 |
| Low security | 4604 | 246 | 1.67 (1.39-2.01) | <0.001 |
| Very low security | 3240 | 177 | 2.26 (1.76-2.91) | <0.001 |
| **Food Security Status** | | | | |
| Food Secure | 28303 | 2136 | 1 (Reference) |  |
| Food Insecure | 12858 | 699 | 1.85 (1.60-2.14) | <0.001 |

HR, hazard ratio;

Multivariate Cox regression models included US population and study design weights to account for the complex survey design. Hazard ratios were adjusted for age, gender, race/ethnicity, education level, annual household income, household size, BMI, disease history of diabetes, hypertension, and hypercholesterolemia, daily energy intake, health insurance coverage, and lifestyles factors, including regular physical activity, current smoking status, current drinking status, and sleep duration

# **eTable 3. Adjusted Hazard Ratios of All-cause Mortality Associated with Different Lifestyles Scores and Food Security Status**

| **Total Score** | **N** | **All-Cause Mortality** | | |
| --- | --- | --- | --- | --- |
|  |  | **Case** | **HR (95% CI)** | **P value** |
| **Total participants** | | | | |
| 0 | 6987 | 484 | 1 (Reference) |  |
| 1 | 17463 | 1186 | 0.74 (0.64-0.86) | <0.001 |
| 2 | 12876 | 905 | 0.69 (0.58-0.82) | <0.001 |
| 3-4 | 3835 | 260 | 0.62 (0.50-0.78) | <0.001 |
| **Food Security population** | | | | |
| 0 | 4556 | 340 | 1 (Reference) |  |
| 1 | 11847 | 866 | 0.72 (0.6-0.86) | <0.001 |
| 2 | 9071 | 722 | 0.64 (0.53-0.78) | <0.001 |
| 3-4 | 2829 | 208 | 0.51 (0.4-0.64) | <0.001 |
| **Food Insecurity population** | | | | |
| 0 | 2431 | 144 | 1 (Reference) |  |
| 1 | 5616 | 320 | 0.60 (0.47-0.77) | <0.001 |
| 2 | 3805 | 183 | 0.46 (0.35-0.60) | <0.001 |
| 3-4 | 1006 | 52 | 0.39 (0.23-0.66) | 0.001 |

HR, hazard ratio;

Multivariate Cox regression models included US population and study design weights to account for the complex survey design. Hazard ratios were adjusted for age, gender, race/ethnicity, education level, annual household income, household size, BMI, disease history of diabetes, hypertension, and hypercholesterolemia, daily energy intake, health insurance coverage, and lifestyles factors, including regular physical activity, current smoking status, current drinking status, and sleep duration. When analyzing individual lifestyle behavior, adjustments were made for other lifestyle behaviors accordingly.

# **eTable 4. Stratified Hazard Ratios for All-Cause Mortality Associated with Individual Lifestyle Factors**

| **Total participants** | **All-cause Mortality** | | | | **Food Security** | **All-cause Mortality** | | | **Food**  **Insecurity** | **All-cause Mortality** | | |
| --- | --- | --- | --- | --- | --- | --- | --- | --- | --- | --- | --- | --- |
|  | **Case/N** | | **HR (95% CI)** | **P value** |  | **Case/N** | **HR (95% CI)** | **P value** |  | **Case/N** | **HR (95% CI)** | **P value** |
| **Smoking Status** | | | | | **Smoking Status** | | | | **Smoking Status** | | | |
| Current | 555/8039 | | 1 (Reference) |  | Current | 327/4422 | 1 (Reference) |  | Current | 228/3617 | 1 (Reference) |  |
| Quitting | 1111/9350 | | 0.66 (0.56-0.78) | <0.001 | Quitting | 893/7007 | 0.70 (0.58-0.86) | 0.001 | Quitting | 218/2343 | 0.72 (0.56-0.93) | 0.011 |
| Never | 1151/22276 | | 0.53 (0.47-0.60) | <0.001 | Never | 907/15955 | 0.58 (0.50-0.68) | <0.001 | Never | 244/6321 | 0.53 (0.42-0.66) | <0.001 |
| **Alcohol consumption** | | | | | **Alcohol consumption** | | | | **Alcohol consumption** | | | |
| Current | 1557/25751 | 1 (Reference) | |  | Current | 1146/17812 | 1 (Reference) |  | Current | 411/7939 | 1 (Reference) |  |
| Quitting | 394/5268 | 0.94 (0.78-1.13) | | 0.507 | Quitting | 289/3446 | 0.92 (0.76-1.11) | 0.396 | Quitting | 105/1822 | 0.90 (0.66-1.24) | 0.263 |
| Never | 344/4002 | 0.87 (0.76-0.99) | | 0.042 | Never | 276/2850 | 0.83 (0.72-0.96) | 0.010 | Never | 68/1152 | 0.85 (0.63-1.13) | 0.539 |
| **Physical Activity Status** | | | | | **Physical Activity Status** | | | | **Physical Activity Status** | | | |
| Sedentary | 436/6107 | | 1 (Reference) |  | Sedentary | 290/3501 | 1 (Reference) |  | Sedentary | 146/2606 | 1 (Reference) |  |
| Moderate | 656/17691 | | 0.50 (0.42-0.59) | <0.001 | Moderate | 507/13170 | 0.49 (0.41-0.59) | <0.001 | Moderate | 149/4521 | 0.62 (0.44-0.85) | 0.004 |
| Vigorous | 1743/17361 | | 0.96 (0.83-1.12) | 0.583 | Vigorous | 1339/11632 | 0.94 (0.80-1.11) | 0.473 | Vigorous | 404/5729 | 0.97 (0.74-1.28) | 0.841 |
| **Sleep duration** | | | | | **Sleep duration** | | | | **Sleep duration** | | | |
| ＜6.5h | 1348/13412 | | 1 (Reference) |  | ＜ 6.5h | 1047/11105 | 1 (Reference) |  | ＜6.5h | 301/5109 | 1 (Reference) |  |
| 6.5 -7.5h | 556/11408 | | 0.73 (0.64-0.83) | <0.001 | 6.5 - 7.5h | 428/8290 | 0.75 (0.65-0.85) | <0.001 | 6.5-7.5h | 128/3118 | 0.72 (0.54-0.95) | 0.022 |
| ＞7.5 | 912/16214 | | 0.91 (0.82-1.02) | 0.093 | ＞ 7.5h | 651/8837 | 0.97 (0.86-1.08) | 0.560 | ＞7.5h | 261/4575 | 0.80 (0.63-1.01) | 0.063 |

HR, hazard ratio;

Multivariate Cox regression models included US population and study design weights to account for the complex survey design. Hazard ratios were adjusted for age, gender, race/ethnicity, education level, annual household income, household size, BMI, disease history of diabetes, hypertension, and hypercholesterolemia, daily energy intake, health insurance coverage, and lifestyles factors, including regular physical activity, current smoking status, current drinking status, and sleep duration. When analyzing individual lifestyle behavior, adjustments were made for other lifestyle behaviors accordingly.

# **eTable 5. All-Cause Mortality Hazard Ratios from Joint Effects of Lifestyle Factors and Food Security Status**

| **Category** | **N** | **Case** | **HR (95% CI)** | **P value** |
| --- | --- | --- | --- | --- |
| **Food Security Status & Smoking Status** |  |  |  |  |
| Food insecure & current-smoking | 3617 | 228 | 1 (Reference) |  |
| Food insecure & quitting-smoking | 2343 | 218 | 0.64 (0.5-0.83) | 0.001 |
| Food insecure & never-smoking | 6321 | 244 | 0.49 (0.39-0.61) | <0.001 |
| Food secure & current-smoking | 4422 | 327 | 0.53 (0.43-0.64) | <0.001 |
| Food secure & quitting-smoking | 7007 | 893 | 0.38 (0.3-0.47) | <0.001 |
| Food secure & never-smoking | 15955 | 907 | 0.31 (0.25-0.38) | <0.001 |
| **Food Security Status & Alcohol consumption** |  |  |  |  |
| Food insecure & current-drinking | 7939 | 411 | 1 (Reference) |  |
| Food insecure & quitting-drinking | 1822 | 105 | 0.8 (0.59-1.11) | 0.178 |
| Food insecure & never-drinking | 1152 | 68 | 0.73 (0.56-0.97) | 0.028 |
| Food secure & current-drinking | 17812 | 1146 | 0.56 (0.47-0.67) | <0.001 |
| Food secure & quitting-drinking | 3446 | 289 | 0.53 (0.45-0.63) | <0.001 |
| Food secure & never-drinking | 2850 | 276 | 0.47 (0.4-0.57) | <0.001 |
| **Food Security Status & Physical Activity Status** |  |  |  |  |
| Food insecure & vigorous activity | 2606 | 146 | 1 (Reference) |  |
| Food insecure & sedentary activity | 5729 | 404 | 0.96 (0.72-1.27) | 0.746 |
| Food insecure & moderate activity | 4521 | 149 | 0.68 (0.49-0.96) | 0.030 |
| Food secure & vigorous activity | 3501 | 290 | 0.63 (0.47-0.85) | 0.003 |
| Food secure & sedentary activity | 11632 | 1339 | 0.6 (0.45-0.8) | 0.001 |
| Food secure & moderate activity | 13170 | 507 | 0.29 (0.21-0.39) | <0.001 |
| **Food Security Status & Sleep Duration** |  |  |  |  |
| Food insecure & >7.5-h sleep duration | 5109 | 301 | 1 (Reference) |  |
| Food insecure & <6.5-h sleep duration | 4575 | 261 | 0.79 (0.63-1.01) | 0.064 |
| Food insecure & ≥6.5-≤7.5-h sleep duration | 3118 | 128 | 0.74 (0.55-0.98) | 0.046 |
| Food secure & >7.5-h sleep duration | 11105 | 1047 | 0.51 (0.42-0.61) | <0.001 |
| Food secure & <6.5-h sleep duration | 8837 | 651 | 0.49 (0.40-0.59) | <0.001 |
| Food secure & ≥6.5-≤7.5-h sleep duration | 8290 | 428 | 0.37 (0.30-0.46) | <0.001 |

HR, hazard ratio;

Multivariate Cox regression models included US population and study design weights to account for the complex survey design. Hazard ratios were adjusted for age, gender, race/ethnicity, education level, annual household income, household size, BMI, disease history of diabetes, hypertension, and hypercholesterolemia, daily energy intake, health insurance coverage, and lifestyles factors, including regular physical activity, current smoking status, current drinking status, and sleep duration. When analyzing individual lifestyle behavior, adjustments were made for other lifestyle behaviors accordingly.

# **eTable 6. All-cause Mortality Hazard Ratios Stratified by Gender**

| **Category** | **Male** | | **Female** | | **Interaction P value** |
| --- | --- | --- | --- | --- | --- |
|  | HR (95% CI) | P value | HR (95% CI) | P value |  |
| **Food Security Status & Smoking Status** | | | | | |
| Food insecure & current-smoking | 1 (Reference) |  | 1 (Reference) |  | 0.204 |
| Food insecure & quitting-smoking | 0.74 (0.52-1.05) | 0.093 | 0.58 (0.38-0.91) | 0.018 |  |
| Food insecure & never-smoking | 0.60 (0.42-0.87) | 0.007 | 0.35 (0.26-0.47) | <0.001 |  |
| Food secure & current-smoking | 0.67 (0.52-0.85) | 0.002 | 0.36 (0.25-0.51) | <0.001 |  |
| Food secure & quitting-smoking | 0.46 (0.34-0.63) | <0.001 | 0.30 (0.21-0.41) | <0.001 |  |
| Food secure & never-smoking | 0.36 (0.26-0.50) | <0.001 | 0.23 (0.18-0.31) | <0.001 |  |
| **Food Security Status & Alcohol consumption** | | | | | |
| Food insecure & current-drinking | 1 (Reference) |  | 1 (Reference) |  | 0.735 |
| Food insecure & quitting-drinking | 0.66 (0.41-1.08) | 0.099 | 0.75 (0.49-1.14) | 0.178 |  |
| Food insecure & never-drinking | 0.64 (0.51-0.80) | <0.001 | 0.67 (0.47-0.95) | 0.025 |  |
| Food secure & current-drinking | 0.96 (0.47-1.94) | 0.9 | 0.37 (0.29-0.47) | <0.001 |  |
| Food secure & quitting-drinking | 0.75 (0.56-1.02) | 0.068 | 0.49 (0.39-0.62) | <0.001 |  |
| Food secure & never-drinking | 0.58 (0.42-0.78) | 0.001 | 0.52 (0.41-0.65) | <0.001 |  |
| **Food Security Status & Physical Activity Status** | | | | | |
| Food insecure & sedentary activity | 1 (Reference) |  | 1 (Reference) |  | 0.154 |
| Food insecure & vigorous activity | 0.92 (0.65-1.31) | 0.657 | 1.35 (0.86-2.14) | 0.191 |  |
| Food insecure & moderate activity | 0.58 (0.42-0.80) | 0.001 | 0.65 (0.52-0.82) | <0.001 |  |
| Food secure & sedentary activity | 0.59 (0.46-0.75) | <0.001 | 0.93 (0.60-1.45) | 0.758 |  |
| Food secure & vigorous activity | 0.64 (0.48-0.87) | 0.005 | 0.72 (0.53-0.99) | 0.042 |  |
| Food secure & moderate activity | 0.32 (0.24-0.42) | <0.001 | 0.28 (0.21-0.37) | <0.001 |  |
| **Food Security Status & Sleep Duration** | | | | | |
| Food insecure & >7.5-h sleep duration | 1 (Reference) |  | 1 (Reference) |  | 0.267 |
| Food insecure & <6.5-h sleep duration | 0.93 (0.68-1.27) | 0.647 | 0.93 (0.56-1.54) | 0.771 |  |
| Food insecure & ≥6.5-≤7.5-h sleep duration | 0.66 (0.47-0.94) | 0.020 | 0.62 (0.42-0.91) | 0.016 |  |
| Food secure & >7.5-h sleep duration | 0.57 (0.43-0.76) | <0.001 | 0.47 (0.35-0.62) | <0.001 |  |
| Food secure & <6.5-h sleep duration | 0.56 (0.40-0.77) | <0.001 | 0.44 (0.32-0.59) | <0.001 |  |
| Food secure & ≥6.5-≤7.5-h sleep duration | 0.41 (0.29-0.57) | <0.001 | 0.34 (0.24-0.48) | <0.001 |  |
| **Total Score** | | | | | |
| 0 | 1 (Reference) |  | 1 (Reference) |  | 0.050 |
| 1 | 0.60 (0.49-0.74) | <0.001 | 0.71 (0.51-0.99) | 0.049 |  |
| 2 | 0.48 (0.38-0.61) | <0.001 | 0.64 (0.45-0.91) | 0.013 |  |
| 3-4 | 0.34 (0.24-0.49) | <0.001 | 0.53 (0.36-0.76) | 0.001 |  |

HR, hazard ratio;Multivariate Cox regression models included US population and study design weights to account for the complex survey design. Hazard ratios were adjusted for age, race/ethnicity, education level, annual household income, household size, BMI, disease history of diabetes, hypertension, and hypercholesterolemia, daily energy intake, health insurance coverage, and lifestyles factors, including regular physical activity, current smoking status, current drinking status, and sleep duration. When analyzing individual lifestyle behavior, adjustments were made for other lifestyle behaviors accordingly.

# **eTable 7. All-cause Mortality Hazard Ratios Stratified by Age**

| **Category** | **Age < 65** | | **Age ≥ 65** | | **Interaction P value** |
| --- | --- | --- | --- | --- | --- |
|  | HR (95% CI) | P value | HR (95% CI) | P value |  |
| **Food Security Status & Smoking Status** | | | | | |
| Food insecure & current-smoking | 1 |  | 1 (Reference) |  | 0.198 |
| Food insecure & quitting-smoking | 0.92 (0.63-1.32) | 0.634 | 0.79 (0.53-1.18) | 0.244 |  |
| Food insecure & never-smoking | 0.54 (0.40-0.72) | <0.001 | 0.55 (0.43-0.69) | <0.001 |  |
| Food secure & current-smoking | 0.66 (0.51-0.86) | 0.003 | 0.72 (0.51-1.04) | 0.076 |  |
| Food secure & quitting-smoking | 0.45 (0.30-0.67) | <0.001 | 0.67 (0.52-0.86) | 0.002 |  |
| Food secure & never-smoking | 0.31 (0.23-0.44) | <0.001 | 0.58 (0.46-0.74) | <0.001 |  |
| **Food Security Status & Alcohol consumption** | | | | | |
| Food insecure & current-drinking | 1 (Reference) |  | 1 (Reference) |  | 0.324 |
| Food insecure & quitting-drinking | 1.08 (0.71-1.63) | 0.72 | 0.95 (0.67-1.34) | 0.755 |  |
| Food insecure & never-drinking | 0.66 (0.41-1.05) | 0.077 | 0.84 (0.63-1.13) | 0.242 |  |
| Food secure & current-drinking | 0.44 (0.25-0.75) | 0.003 | 0.93 (0.64-1.34) | 0.679 |  |
| Food secure & quitting-drinking | 0.56 (0.36-0.87) | 0.01 | 0.86 (0.65-1.14) | 0.296 |  |
| Food secure & never-drinking | 0.29 (0.20-0.42) | <0.001 | 0.65 (0.49-0.87) | 0.004 |  |
| **Food Security Status & Physical Activity Status** | | | | | |
| Food insecure & sedentary activity | 1 (Reference) |  | 1 (Reference) |  | 0.043 |
| Food insecure & vigorous activity | 0.96 (0.63-1.47) | 0.841 | 1.14 (0.81-1.62) | 0.441 |  |
| Food insecure & moderate activity | 0.48 (0.36-0.64) | <0.001 | 0.75 (0.54-0.98) | 0.047 |  |
| Food secure & sedentary activity | 0.58 (0.43-0.79) | 0.001 | 0.78 (0.64-0.95) | 0.014 |  |
| Food secure & vigorous activity | 0.46 (0.26-0.79) | 0.006 | 0.83 (0.67-1.03) | 0.092 |  |
| Food secure & moderate activity | 0.26 (0.20-0.34) | <0.001 | 0.39 (0.31-0.48) | <0.001 |  |
| **Food Security Status & Sleep Duration** | | | | | |
| Food insecure & >7.5-h sleep duration | 1 (Reference) |  | 1 (Reference) |  | 0.007 |
| Food insecure & <6.5-h sleep duration | 0.97 (0.72-1.29) | 0.814 | 0.66 (0.45-0.96) | 0.030 |  |
| Food insecure & ≥6.5-≤7.5-h sleep duration | 0.82 (0.56-1.19) | 0.289 | 0.58 (0.43-0.79) | 0.001 |  |
| Food secure & >7.5-h sleep duration | 0.58 (0.43-0.79) | 0.001 | 0.55 (0.44-0.68) | <0.001 |  |
| Food secure & <6.5-h sleep duration | 0.51 (0.38-0.68) | <0.001 | 0.55 (0.43-0.68) | <0.001 |  |
| Food secure & ≥6.5-≤7.5-h sleep duration | 0.39 (0.28-0.54) | <0.001 | 0.45 (0.36-0.57) | <0.001 |  |
| **Total Score** | | | | | |
| 0 | 1 (Reference) |  | 1 (Reference) |  | 0.013 |
| 1 | 0.51 (0.37-0.69) | <0.001 | 0.78 (0.67-0.92) | 0.003 |  |
| 2 | 0.38 (0.27-0.54) | <0.001 | 0.69 (0.57-0.85) | 0.001 |  |
| 3-4 | 0.26 (0.17-0.40) | <0.001 | 0.57 (0.46-0.71) | <0.001 |  |

HR, hazard ratio; Multivariate Cox regression models included US population and study design weights to account for the complex survey design. Hazard ratios were adjusted for gender, race/ethnicity, education level, annual household income, household size, BMI, disease history of diabetes, hypertension, and hypercholesterolemia, daily energy intake, health insurance coverage, and lifestyles factors, including regular physical activity, current smoking status, current drinking status, and sleep duration. When analyzing individual lifestyle behavior, adjustments were made for other lifestyle behaviors accordingly.

# **eTable 8. All-cause Mortality Hazard Ratios Stratified by Health Insurance Status**

| **Category** | **Insured** | | **Uninsured** | | **Interaction P value** |
| --- | --- | --- | --- | --- | --- |
|  | HR (95% CI) | P value | HR (95% CI) | P value |  |
| **Food Security Status & Smoking Status** | | | | | |
| Food insecure & current-smoking | 1 (Reference) |  | 1 (Reference) |  | 0.204 |
| Food insecure & quitting-smoking | 0.64 (0.49-0.85) | 0.002 | 0.47 (0.25-0.88) | 0.019 |  |
| Food insecure & never-smoking | 0.46 (0.36-0.58) | <0.001 | 0.58 (0.34-0.99) | 0.048 |  |
| Food secure & current-smoking | 0.48 (0.38-0.60) | <0.001 | 0.86 (0.53-1.40) | 0.545 |  |
| Food secure & quitting-smoking | 0.34 (0.27-0.43) | <0.001 | 0.60 (0.35-1.05) | 0.074 |  |
| Food secure & never-smoking | 0.29 (0.23-0.36) | <0.001 | 0.46 (0.28-0.75) | 0.002 |  |
| **Food Security Status & Alcohol consumption** | | | | | |
| Food insecure & current-drinking | 1 (Reference) |  | 1 (Reference) |  | 0.938 |
| Food insecure & quitting-drinking | 0.90 (0.65-1.26) | 0.552 | 0.82 (0.55-1.22) | 0.322 |  |
| Food insecure & never-drinking | 0.76 (0.57-1.02) | 0.066 | 0.79 (0.42-1.51) | 0.480 |  |
| Food secure & current-drinking | 0.45 (0.36-0.56) | <0.001 | 0.64 (0.31-1.34) | 0.232 |  |
| Food secure & quitting-drinking | 0.54 (0.43-0.68) | <0.001 | 0.51 (0.22-1.20) | 0.123 |  |
| Food secure & never-drinking | 0.53 (0.43-0.65) | <0.001 | 0.29 (0.10-0.87) | 0.027 |  |
| **Food Security Status & Physical Activity Status** | | | | | |
| Food insecure & sedentary activity | 1 (Reference) |  | 1 (Reference) |  | 0.325 |
| Food insecure & vigorous activity | 0.88 (0.66-1.17) | 0.37 | 0.51 (0.22-1.17) | 0.110 |  |
| Food insecure & moderate activity | 0.62 (0.42-0.90) | 0.012 | 0.55 (0.35-0.88) | 0.012 |  |
| Food secure & sedentary activity | 0.56 (0.40-0.78) | 0.001 | 0.94 (0.62-1.44) | 0.772 |  |
| Food secure & vigorous activity | 0.53 (0.38-0.72) | <0.001 | 0.94 (0.39-2.25) | 0.886 |  |
| Food secure & moderate activity | 0.25 (0.18-0.35) | <0.001 | 0.45 (0.28-0.70) | 0.001 |  |
| **Food Security Status & Sleep Duration** | | | | | |
| Food insecure & >7.5-h sleep duration | 1 (Reference) |  | 0.76 (0.5-1.14) | 0.176 | 0.035 |
| Food insecure & <6.5-h sleep duration | 0.75 (0.58-0.97) | 0.029 | 0.95 (0.6-1.52) | 0.830 |  |
| Food insecure & ≥6.5-≤7.5-h sleep duration | 0.68 (0.48-0.95) | 0.024 | 0.90 (0.54-1.49) | 0.669 |  |
| Food secure & >7.5-h sleep duration | 0.47 (0.38-0.57) | <0.001 | 1 (Reference) |  |  |
| Food secure & <6.5-h sleep duration | 0.44 (0.36-0.55) | <0.001 | 0.80 (0.50-1.27) | 0.345 |  |
| Food secure & ≥6.5-≤7.5-h sleep duration | 0.35 (0.28-0.43) | <0.001 | 0.58 (0.35-0.95) | 0.029 |  |
| **Total Score** | | | | | |
| 0 | 1 (Reference) |  | 1 (Reference) |  |  |
| 1 | 0.70 (0.61-0.80) | <0.001 | 0.57 (0.31-1.04) | 0.066 |  |
| 2 | 0.59 (0.50-0.70) | <0.001 | 0.49 (0.26-0.94) | 0.033 |  |
| 3-4 | 0.47 (0.38-0.59) | <0.001 | 0.26 (0.11-0.64) | 0.004 |  |

HR, hazard ratio; Multivariate Cox regression models included US population and study design weights to account for the complex survey design. Hazard ratios were adjusted for age, gender, race/ethnicity, education level, annual household income, household size, BMI, disease history of diabetes, hypertension, and hypercholesterolemia, daily energy intake, and lifestyles factors, including regular physical activity, current smoking status, current drinking status, and sleep duration. When analyzing individual lifestyle behavior, adjustments were made for other lifestyle behaviors accordingly.

**eTable 9. Multiplicative interaction tests between Food Security Status and lifestyle factors (and the composite lifestyle score) for all-cause mortality**

| Category | HR (95%CI) for interaction | P value for interaction |
| --- | --- | --- |
| Food Security Status & Smoking Status | 1.28 (1.01-1.62) | 0.041 |
| Food Security Status & Alcohol consumption | 1.41 (1.03-1.93) | 0.032 |
| Food Security Status & Physical Activity Status | 1.43 (1.16-1.77) | 0.001 |
| Food Security Status & Sleep Duration | 1.22 (1.12-1.32) | <0.001 |
| Food Security Status & lifestyle score | 1.23 (1.08-1.41) | 0.003 |

HR, hazard ratio;

# **eTable 10. Hazard Ratios for All-Cause Mortality by Food Security Status and Lifestyle After Excluding Participants with Follow-Up Less Than 2 Years**

| **Category** | **N** | **Case** | **HR (95% CI)** | **P value** |
| --- | --- | --- | --- | --- |
| **Food Security Status & Smoking Status** | | | | |
| Food insecure & current-smoking | 3018 | 148 | 1 (Reference) |  |
| Food insecure & quitting-smoking | 1902 | 127 | 0.54 (0.40-0.74) | <0.001 |
| Food insecure & never-smoking | 5368 | 173 | 0.45 (0.34-0.61) | <0.001 |
| Food secure & current-smoking | 3794 | 228 | 0.51 (0.39-0.66) | <0.001 |
| Food secure & quitting-smoking | 8921 | 638 | 0.35 (0.27-0.46) | <0.001 |
| Food secure & never-smoking | 13567 | 612 | 0.29 (0.23-0.37) | <0.001 |
| **Food Security Status & Alcohol consumption** | | | | |
| Food insecure & current-drinking | 6553 | 264 | 1 (Reference) |  |
| Food insecure & quitting-drinking | 1045 | 48 | 0.75 (0.54-1.06) | 0.098 |
| Food insecure & never-drinking | 1547 | 80 | 0.72 (0.51-0.99) | 0.048 |
| Food secure & current-drinking | 15003 | 824 | 0.56 (0.44-0.71) | <0.001 |
| Food secure & quitting-drinking | 2580 | 205 | 0.50 (0.40-0.64) | <0.001 |
| Food secure & never-drinking | 2918 | 197 | 0.47 (0.37-0.58) | <0.001 |
| **Food Security Status & Physical Activity Status** | | | | |
| Food insecure & sedentary activity | 4929 | 282 | 1 (Reference) |  |
| Food insecure & vigorous activity | 1973 | 63 | 0.84 (0.62-1.14) | 0.266 |
| Food insecure & moderate activity | 3961 | 112 | 0.74 (0.56-0.98) | 0.047 |
| Food secure & sedentary activity | 2607 | 163 | 0.69 (0.54-0.88) | 0.004 |
| Food secure & vigorous activity | 9977 | 924 | 0.6 (0.49-0.74) | <0.001 |
| Food secure & moderate activity | 11613 | 398 | 0.32 (0.26-0.41) | <0.001 |
| **Food Security Status & Sleep Duration** | | | | |
| Food insecure & >7.5-h sleep duration | 4255 | 194 | 1 (Reference) |  |
| Food insecure & <6.5-h sleep duration | 3927 | 178 | 0.74 (0.57-0.97) | 0.026 |
| Food insecure & ≥6.5-≤7.5-h sleep duration | 2641 | 82 | 0.68 (0.49-0.95) | 0.023 |
| Food secure & >7.5-h sleep duration | 9313 | 708 | 0.49 (0.39-0.61) | <0.001 |
| Food secure & <6.5-h sleep duration | 7738 | 463 | 0.47 (0.36-0.6) | <0.001 |
| Food secure & ≥6.5-≤7.5-h sleep duration | 7092 | 310 | 0.36 (0.28-0.46) | <0.001 |
| **Total Score** | | | | |
| 0 | 5916 | 322 | 1 (Reference) |  |
| 1 | 14754 | 807 | 0.67 (0.56-0.80) | <0.001 |
| 2 | 11026 | 623 | 0.56 (0.45-0.69) | <0.001 |
| 3-4 | 3366 | 190 | 0.48 (0.37-0.63) | <0.001 |

HR, hazard ratio; Multivariate Cox regression models included US population and study design weights to account for the complex survey design. Hazard ratios were adjusted for age, gender, race/ethnicity, education level, annual household income, household size, BMI, disease history of diabetes, hypertension, and hypercholesterolemia, daily energy intake, health insurance coverage, and lifestyles factors, including regular physical activity, current smoking status, current drinking status, and sleep duration. When analyzing individual lifestyle behavior, adjustments were made for other lifestyle behaviors accordingly.

# **eTable 11. Hazard ratios for All-Cause Mortality by Food Security Status and Lifestyle After Excluding Chronic Diseases**

| **Category** | N | **Case** | **HR (95% CI)** | **P value** |
| --- | --- | --- | --- | --- |
| **Food Security Status & Smoking Status** | | | | |
| Food insecure & current-smoking | 626 | 30 | 1 (Reference) |  |
| Food insecure & quitting-smoking | 1354 | 40 | 0.84 (0.47-1.51) | 0.557 |
| Food insecure & never-smoking | 2719 | 37 | 0.67 (0.35-1.25) | 0.205 |
| Food secure & current-smoking | 1856 | 148 | 0.50 (0.30-0.83) | 0.008 |
| Food secure & quitting-smoking | 1635 | 54 | 0.38 (0.20-0.70) | 0.002 |
| Food secure & never-smoking | 6813 | 152 | 0.28 (0.16-0.48) | <0.001 |
| **Food Security Status & Alcohol consumption** | | | | |
| Food insecure & current-drinking | 3005 | 75 | 1 (Reference) |  |
| Food insecure & quitting-drinking | 386 | 7 | 0.64 (0.27-1.48) | 0.29 |
| Food insecure & never-drinking | 716 | 10 | 0.68 (0.24-1.96) | 0.47 |
| Food secure & current-drinking | 6672 | 198 | 0.38 (0.26-0.56) | <0.001 |
| Food secure & quitting-drinking | 915 | 35 | 0.48 (0.28-0.82) | 0.008 |
| Food secure & never-drinking | 1280 | 42 | 0.31 (0.18-0.54) | <0.001 |
| **Food Security Status & Physical Activity Status** | | | | |
| Food insecure & sedentary activity | 1758 | 60 | 1 (Reference) |  |
| Food insecure & vigorous activity | 1094 | 25 | 0.42 (0.21-0.87) | 0.02 |
| Food insecure & moderate activity | 1854 | 22 | 0.45 (0.25-0.81) | 0.009 |
| Food secure & sedentary activity | 3436 | 198 | 0.29 (0.18-0.47) | <0.001 |
| Food secure & vigorous activity | 1412 | 50 | 0.28 (0.15-0.52) | <0.001 |
| Food secure & moderate activity | 5463 | 107 | 0.18 (0.11-0.29) | <0.001 |
| **Food Security Status & Sleep Duration** | | | | |
| Food insecure & >7.5-h sleep duration | 1986 | 44 | 1 (Reference) |  |
| Food insecure & <6.5-h sleep duration | 1455 | 35 | 0.92 (0.52-1.64) | 0.776 |
| Food insecure & ≥6.5-≤7.5-h sleep duration | 1250 | 27 | 0.91 (0.45-1.84) | 0.787 |
| Food secure & >7.5-h sleep duration | 4059 | 181 | 0.36 (0.23-0.58) | <0.001 |
| Food secure & <6.5-h sleep duration | 2948 | 90 | 0.34 (0.20-0.57) | <0.001 |
| Food secure & ≥6.5-≤7.5-h sleep duration | 3285 | 81 | 0.30 (0.18-0.5) | <0.001 |
| **Total Score** | | | | |
| 0 | 2180 | 91 | 1 (Reference) |  |
| 1 | 6557 | 185 | 0.95 (0.68-1.34) | 0.777 |
| 2 | 4946 | 150 | 0.75 (0.45-1.25) | 0.265 |
| 3-4 | 1334 | 36 | 0.61 (0.45-0.84) | 0.003 |

HR, hazard ratio; Multivariate Cox regression models included US population and study design weights to account for the complex survey design. Hazard ratios were adjusted for age, gender, race/ethnicity, education level, annual household income, household size, BMI, disease history of diabetes, hypertension, and hypercholesterolemia, daily energy intake, health insurance coverage, and lifestyles factors, including regular physical activity, current smoking status, current drinking status, and sleep duration. When analyzing individual lifestyle behavior, adjustments were made for other lifestyle behaviors accordingly.

# **eTable 12. Hazard ratios for All-Cause Mortality by Food Security Status and Lifestyle After Additional Adjustment for Sleep-related Factors**

| **Category** | **HR (95% CI)** | **P value** |
| --- | --- | --- |
| **Food Security Status & Smoking Status** | | |
| Food insecure & current-smoking | 1 (Reference) |  |
| Food insecure & quitting-smoking | 0.64 (0.50-0.83) | 0.001 |
| Food insecure & never-smoking | 0.49 (0.40-0.62) | <0.001 |
| Food secure & current-smoking | 0.54 (0.44-0.66) | <0.001 |
| Food secure & quitting-smoking | 0.38 (0.31-0.48) | <0.001 |
| Food secure & never-smoking | 0.32 (0.26-0.39) | <0.001 |
| **Food Security Status & Alcohol consumption** | | |
| Food insecure & current-drinking | 1 (Reference) |  |
| Food insecure & quitting-drinking | 0.81 (0.59-1.12) | 0.202 |
| Food insecure & never-drinking | 0.75 (0.57-0.99) | 0.048 |
| Food secure & current-drinking | 0.48 (0.41-0.58) | <0.001 |
| Food secure & quitting-drinking | 0.58 (0.48-0.69) | <0.001 |
| Food secure & never-drinking | 0.56 (0.47-0.66) | <0.001 |
| **Food Security Status & Physical Activity Status** | | |
| Food insecure & sedentary activity | 1 (Reference) |  |
| Food insecure & vigorous activity | 0.96 (0.73-1.27) | 0.762 |
| Food insecure & moderate activity | 0.69 (0.49-0.97) | 0.033 |
| Food secure & sedentary activity | 0.64 (0.47-0.86) | 0.003 |
| Food secure & vigorous activity | 0.61 (0.46-0.82) | 0.001 |
| Food secure & moderate activity | 0.30 (0.22-0.40) | <0.001 |
| **Food Security Status & Sleep Duration** | | |
| Food insecure & >7.5-h sleep duration | 1 (Reference) |  |
| Food insecure & <6.5-h sleep duration | 0.77 (0.60-0.98) | <0.001 |
| Food insecure & ≥6.5-≤7.5-h sleep duration | 0.75 (0.56-0.99) | 0.008 |
| Food secure & >7.5-h sleep duration | 0.53 (0.44-0.64) | 0.001 |
| Food secure & <6.5-h sleep duration | 0.48 (0.40-0.59) | 0.013 |
| Food secure & ≥6.5-≤7.5-h sleep duration | 0.38 (0.31-0.47) | 0.049 |
| **Total Score** | | |
| 0 | 1 (Reference) |  |
| 1 | 0.68 (0.59-0.79) | <0.001 |
| 2 | 0.58 (0.50-0.69) | <0.001 |
| 3-4 | 0.46 (0.37-0.57) | <0.001 |

HR, hazard ratio; Multivariate Cox regression models included US population and study design weights to account for the complex survey design. Hazard ratios were adjusted for age, gender, race/ethnicity, education level, annual household income, household size, BMI, disease history of diabetes, hypertension, and hypercholesterolemia, daily energy intake, health insurance coverage, and lifestyles factors, including regular physical activity, current smoking status, current drinking status, and sleep duration. When analyzing individual lifestyle behavior, adjustments were made for other lifestyle behaviors accordingly.

# **eTable 13. Hazard ratios for All-Cause Mortality by Food Security Status and Lifestyle After Additional Adjustment for Energy-related Factors**

| **Category** | N | **Case** | **HR (95% CI)** | **P value** |
| --- | --- | --- | --- | --- |
| **Food Security Status & Smoking Status** | | | | |
| Food insecure & current-smoking | 3526 | 221 | 1 (Reference) |  |
| Food insecure & quitting-smoking | 2319 | 215 | 0.65 (0.50-0.86) | 0.002 |
| Food insecure & never-smoking | 6251 | 238 | 0.46 (0.36-0.58) | <0.001 |
| Food secure & current-smoking | 4352 | 318 | 0.53 (0.42-0.67) | <0.001 |
| Food secure & quitting-smoking | 6969 | 889 | 0.38 (0.29-0.48) | <0.001 |
| Food secure & never-smoking | 15834 | 900 | 0.30 (0.24-0.37) | <0.001 |
| **Food Security Status & Alcohol consumption** | | | | |
| Food insecure & current-drinking | 7809 | 402 | 1 (Reference) |  |
| Food insecure & quitting-drinking | 1137 | 64 | 0.71 (0.49-1.05) | 0.084 |
| Food insecure & never-drinking | 1796 | 104 | 0.72 (0.53-0.99) | 0.045 |
| Food secure & current-drinking | 17651 | 1135 | 0.47 (0.38-0.58) | <0.001 |
| Food secure & quitting-drinking | 2830 | 273 | 0.55 (0.45-0.67) | <0.001 |
| Food secure & never-drinking | 3411 | 286 | 0.52 (0.41-0.64) | <0.001 |
| **Food Security Status & Physical Activity Status** | | | | |
| Food insecure & sedentary activity | 2565 | 142 | 1 (Reference) |  |
| Food insecure & vigorous activity | 5654 | 394 | 0.90 (0.65-1.26) | 0.552 |
| Food insecure & moderate activity | 4445 | 147 | 0.68 (0.47-0.98) | 0.037 |
| Food secure & sedentary activity | 3470 | 290 | 0.64 (0.46-0.89) | 0.008 |
| Food secure & vigorous activity | 11533 | 1323 | 0.57 (0.42-0.79) | 0.001 |
| Food secure & moderate activity | 13059 | 503 | 0.29 (0.21-0.40) | <0.001 |
| **Food Security Status & Sleep Duration** | | | | |
| Food insecure & >7.5-h sleep duration | 5038 | 292 | 1 (Reference) |  |
| Food insecure & <6.5-h sleep duration | 4497 | 256 | 0.75 (0.59-0.96) | 0.022 |
| Food insecure & ≥6.5-≤7.5-h sleep duration | 3077 | 126 | 0.83 (0.62-1.12) | 0.223 |
| Food secure & >7.5-h sleep duration | 11024 | 1040 | 0.51 (0.42-0.63) | <0.001 |
| Food secure & <6.5-h sleep duration | 8728 | 641 | 0.49 (0.39-0.61) | <0.001 |
| Food secure & ≥6.5-≤7.5-h sleep duration | 8241 | 426 | 0.39 (0.30-0.49) | <0.001 |
| **Total Score** | | | | |
| 0 | 6884 | 478 | 1 (Reference) |  |
| 1 | 17261 | 1171 | 0.69 (0.58-0.82) | <0.001 |
| 2 | 12775 | 894 | 0.57 (0.47-0.69) | <0.001 |
| 3-4 | 3807 | 256 | 0.46 (0.37-0.59) | <0.001 |

HR, hazard ratio; Multivariate Cox regression models included US population and study design weights to account for the complex survey design. Hazard ratios were adjusted for age, gender, race/ethnicity, education level, annual household income, household size, BMI, disease history of diabetes, hypertension, and hypercholesterolemia, daily energy intake, health insurance coverage, and lifestyles factors, including regular physical activity, current smoking status, current drinking status, and sleep duration. When analyzing individual lifestyle behavior, adjustments were made for other lifestyle behaviors accordingly.

# **eTable 14. Hazard ratios for All-Cause Mortality by Food Security Status and Lifestyle After Additional Adjustment for** diet quality using the Healthy Eating Index-2015

| **Category** | **HR (95% CI)** | **P value** |
| --- | --- | --- |
| **Food Security Status & Smoking Status** | | |
| Food insecure & current-smoking | 1 (Reference) |  |
| Food insecure & quitting-smoking | 0.61 (0.48-0.80) | <0.001 |
| Food insecure & never-smoking | 0.46 (0.37-0.58) | <0.001 |
| Food secure & current-smoking | 0.52 (0.42-0.64) | <0.001 |
| Food secure & quitting-smoking | 0.35 (0.29-0.44) | <0.001 |
| Food secure & never-smoking | 0.29 (0.24-0.36) | <0.001 |
| **Food Security Status & Alcohol consumption** | | |
| Food insecure & current-drinking | 1 (Reference) |  |
| Food insecure & quitting-drinking | 0.78 (0.56-1.07) | 0.120 |
| Food insecure & never-drinking | 0.73 (0.55-0.97) | 0.032 |
| Food secure & current-drinking | 0.46 (0.38-0.55) | <0.001 |
| Food secure & quitting-drinking | 0.55 (0.46-0.65) | <0.001 |
| Food secure & never-drinking | 0.53 (0.44-0.63) | <0.001 |
| **Food Security Status & Physical Activity Status** | | |
| Food insecure & sedentary activity | 1 (Reference) |  |
| Food insecure & vigorous activity | 0.92 (0.71-1.19) | 0.513 |
| Food insecure & moderate activity | 0.65 (0.47-0.91) | 0.011 |
| Food secure & sedentary activity | 0.61 (0.46-0.82) | 0.001 |
| Food secure & vigorous activity | 0.58 (0.44-0.76) | <0.001 |
| Food secure & moderate activity | 0.28 (0.21-0.38) | <0.001 |
| **Food Security Status & Sleep Duration** | | |
| Food insecure & >7.5-h sleep duration | 1 (Reference) |  |
| Food insecure & <6.5-h sleep duration | 0.75 (0.56-0.99) | 0.042 |
| Food insecure & ≥6.5-≤7.5-h sleep duration | 0.83 (0.66-1.06) | 0.128 |
| Food secure & >7.5-h sleep duration | 0.52 (0.43-0.62) | <0.001 |
| Food secure & <6.5-h sleep duration | 0.50 (0.41-0.61) | <0.001 |
| Food secure & ≥6.5-≤7.5-h sleep duration | 0.37 (0.30-0.46) | <0.001 |
| **Total Score** | | |
| 0 | 1 (Reference) |  |
| 1 | 0.69 (0.58-0.82) | <0.001 |
| 2 | 0.57 (0.47-0.69) | <0.001 |
| 3-4 | 0.46 (0.37-0.59) | <0.001 |

HR, hazard ratio; Multivariate Cox regression models included US population and study design weights to account for the complex survey design. Hazard ratios were adjusted for age, gender, race/ethnicity, education level, annual household income, household size, BMI, disease history of diabetes, hypertension, and hypercholesterolemia, daily energy intake, health insurance coverage, and lifestyles factors, including regular physical activity, current smoking status, current drinking status, and sleep duration. When analyzing individual lifestyle behavior, adjustments were made for other lifestyle behaviors accordingly.


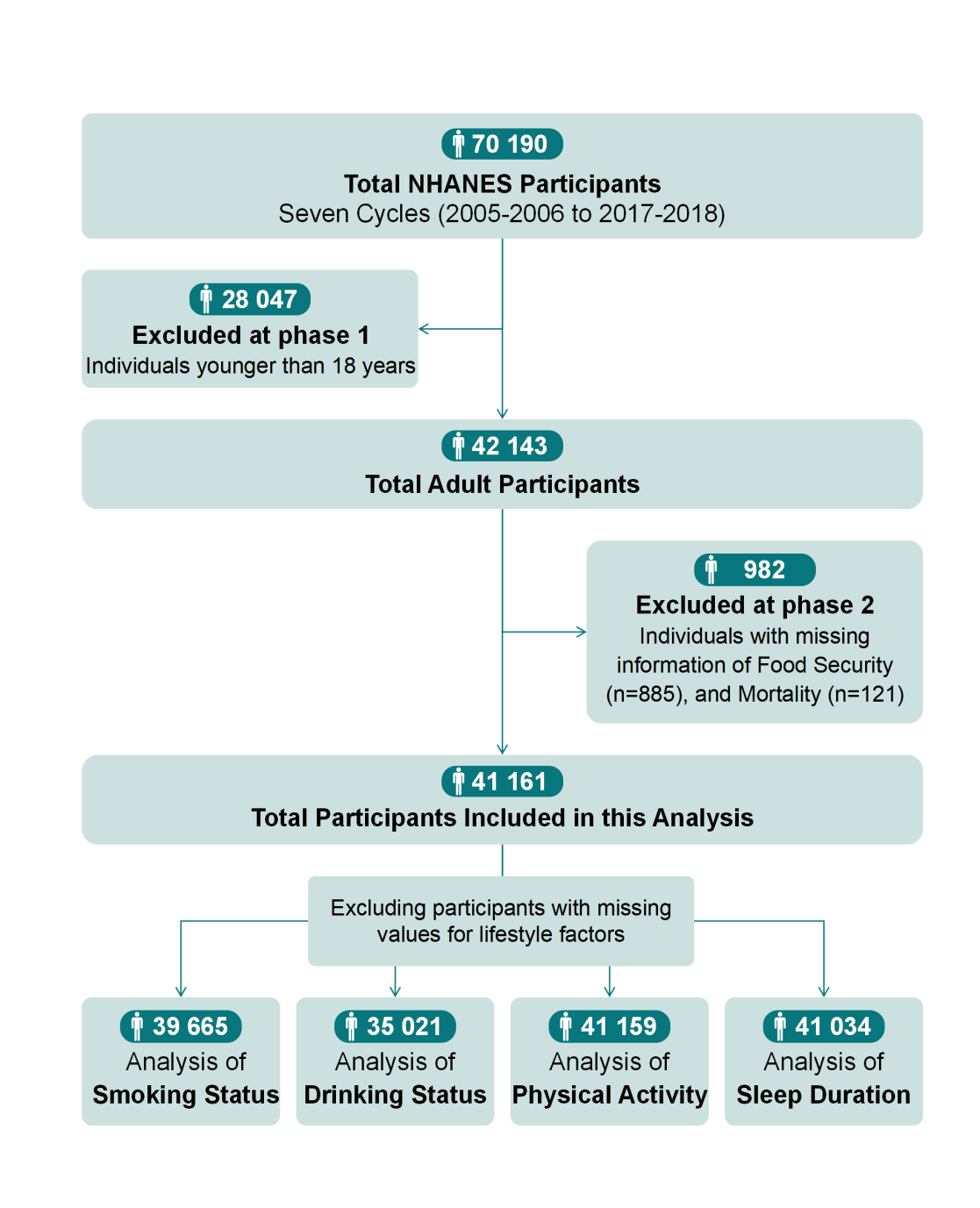


# **eFigure 1. Flow Chart of Participants Enrolment**
